# Burn-Related Glycocalyx Derangement and the Emerging Role of MMP8 in Syndecan Shedding

**DOI:** 10.3390/biology14030269

**Published:** 2025-03-06

**Authors:** Hannes Kühtreiber, Daniel Bormann, Melanie Salek, Lisa Auer, Thomas Haider, Caterina Selina Mildner, Marie-Therese Lingitz, Clemens Aigner, Christine Radtke, Daniel Zimpfer, Hendrik Jan Ankersmit, Michael Mildner

**Affiliations:** 1 Department of Thoracic Surgery, Applied Immunology Laboratory, Medical University of Vienna, 1090 Vienna, Austria; 2 Aposcience AG, 1200 Vienna, Austria; 3 Department of Dermatology, Medical University of Vienna, 1090 Vienna, Austria; 4 Comprehensive Center for Chest Diseases, Medical University of Vienna, 1090 Vienna, Austria; 5 Department of Neurology, Medical University of Vienna, 1090 Vienna, Austria; 6 Department of Orthopedics and Trauma Surgery, Medical University of Vienna, 1090 Vienna, Austria; 7 Division of General Anesthesia and Intensive Care Medicine, Department of Anesthesia, Critical Care and Pain Medicine, Medical University of Vienna, 1090 Vienna, Austria; 8 Department of Plastic, Reconstructive and Aesthetic Surgery, Medical University of Vienna, 1090 Vienna, Austria; 9 Department of Cardiac Surgery, Medical University of Vienna, 1090 Vienna, Austria

**Keywords:** burn injury, glycocalyx, MMP8, ARDS

## Abstract

Burn injuries often lead to severe complications, including acute respiratory distress syndrome (ARDS), driven in part by systemic inflammation and glycocalyx disruption. In this study, we analyzed the sera of 28 patients after burn trauma and utilized single-cell RNA sequencing (scRNA-seq) along with microarray transcriptomic analysis to decipher the impact of burn injury on glycocalyx derangement. We observed the significant upregulation of immune cell-derived degrading enzymes, particularly matrix metalloproteinase-8 (MMP8), which correlated with increased immune cell infiltration and glycocalyx derangement. Serum analyses of burn patients revealed significantly elevated levels of shed glycocalyx components and MMP8, both correlating with the presence of inhalation injury. Consequently, the treatment of human in vitro lung tissue models with MMP8 induced significant glycocalyx shedding in alveolar epithelial cells. Together, based on these findings, we propose that MMP8 plays a previously unrecognized role in glycocalyx disruption and subsequent lung injury post-burn, which implies that inhibiting MMP8 may represent a promising therapeutic strategy for alleviating lung injury after burn trauma.

## Simple Summary

Burn injuries often lead to severe complications, such as organ failure and respiratory failure, driven in part by systemic inflammation and the disruption of the glycocalyx—a carbohydrate-rich layer covering cells. This dynamic matrix plays a crucial role in diverse physiological functions, including maintaining the microcirculation barrier, regulating inflammation and coagulation, facilitating cell–cell communication, and supporting surfactant function in the lungs. When disrupted, as in burn trauma, the glycocalyx’s components are shed, altering its properties and contributing to widespread tissue damage. We investigated factors involved in glycocalyx derangement post-burn trauma across species and identified the enzyme matrix metalloproteinase-8, derived mainly from neutrophils and, to a lesser extent, from monocytes and macrophages, as being significantly increased after burns. Notably, matrix metalloproteinase-8 regulation was conserved across species and correlated with elevated syndecan-1 levels, a key glycocalyx component, in burn patients. Furthermore, we demonstrated in vitro that matrix metalloproteinase-8 cleaves syndecan-1 and, to a lesser extent, syndecan-4. This cleavage may contribute to pulmonary complications post-burn, as glycocalyx integrity is essential for respiratory function. Future research on targeted matrix metalloproteinase-8 inhibition could reduce glycocalyx degradation and help mitigate respiratory and other related complications, supporting recovery in burn patients.

## 1. Introduction

Trauma associated with burn injuries covers a wide range, including exposure to extreme temperatures, harmful radiation, chemical exposures, and intense friction [1]. Frequently underestimated, these injuries predominantly impact the skin, central airways or lungs and are associated with high morbidity and mortality [2]. Several factors influence the clinical management of burn injuries, including their severity and cause, the occurrence of respiratory injury, the timing of medical intervention, and individual factors such as comorbidities and age [3]. Management typically includes fluid resuscitation [4], wound care, and potential surgical interventions such as debridement or skin grafting [5], along with pain management and continuous rehabilitation to support both physical and psychological recovery [6]. Despite advancements in burn care, patient outcomes are still profoundly affected by severe complications such as multiple organ failure, sepsis, and respiratory failure, all of which are associated with a high mortality rate [2].

Within hours post-injury, burns trigger severe disruptions in the innate and adaptive immune system. The complex dysregulation is marked by an early activation of innate immune cells and the release of pro-inflammatory cytokines and chemokines, including interleukin-1 (IL-1), interleukin-6 (IL-6), interleukin-8 (IL-8), and tumor necrosis factor-alpha (TNFα), among others [7]. The initial systemic inflammation response eventually transitions to an anti-inflammatory environment. This shift is facilitated by immunosuppressive factors, notably interleukin 10 (IL-10) and transforming growth factor beta (TGFβ1) [8].

We have previously shown that burn injuries lead to elevated serum levels of soluble suppression of tumorigenicity 2 (sST2), which predict mortality [9], and an increase in neutrophil extracellular trap formation (NETosis)-related factors, indicating systemic neutrophil activation [10]. While crucial in the innate host defense against pathogens and in debridement, neutrophils lead to substantial collateral tissue damage when they become overactivated post-burn. Once systemically activated, polymorphonuclear leukocytes migrate into the lung [11] and induce tissue damage through NETosis [12], degranulation, and reactive oxygen species (ROS) production, among other factors [13]. This inflammatory setting post-burn leads to the release of various proteolytic enzymes including MMPs, neutrophil elastase, and myeloperoxidase, among others, collectively harming glycocalyx integrity, thereby contributing to complications such as acute respiratory distress syndrome (ARDS) [14,15,16]. MMP8, a member of the MMP family and also referred to as neutrophil collagenase, is a collagenolytic enzyme primarily synthesized by neutrophils [17]. However, its specific role in glycocalyx degradation remains undetermined. Notably, research has shown that 24% of burn patients develop ARDS, and of those, 31% succumb to the condition [18]. ARDS typically develops within the first week post-burn, with its severity directly correlating with the total body surface area (TBSA) affected, the duration of mechanical ventilation, and an increased risk of mortality [19]. Inhalation injury [20], systemic inflammation, protein loss, and fluid resuscitation [21] further put patients at an increased risk of respiratory failure.

Recent studies have emphasized the crucial role of the glycocalyx in the development of ARDS [22]. The glycocalyx, a dynamic pericellular matrix, is composed primarily of proteoglycans to which glycosaminoglycans (GAGs) are attached [23]. Specific proteoglycans like mimecan and perlecan are naturally secreted, while transmembrane syndecans and glycosylphosphatidylinositol (GPI)-anchored glypicans play a vital role in securing the glycocalyx to the cell surface [24]. This delicate matrix is subject to continuous remodeling, with degradation frequently mediated by mechanical stress, matrix metalloproteinases (MMPs) [25], and reactive oxygen species (ROS), among other factors, in response to pathological conditions and inflammation [26], which can profoundly impact vascular and lung tissue integrity [22,27].

It could be shown that elevated serum levels of syndecan-1 (SDC1) are linked to a heightened risk of developing ARDS in patients with septic shock [14] and non-pulmonary sepsis [28]. Given the considerable research interest in the glycocalyx of the lung’s endothelial cells, the glycocalyx of the alveolar epithelium has remained underemphasized in comparison. The alveolar glycocalyx, which connects the epithelium to the surfactant layer, is critical in sustaining normal lung function [15,22]. Following direct lung injury, the shedding of the alveolar epithelial glycocalyx has been associated with surfactant dysfunction, correlating with both the duration and severity of respiratory complications [22]. The subsequent impairment of surfactant function was found to result in atelectasis and decreased lung compliance [29].

In this study, we aim to investigate the impact of burn injury on glycocalyx disruption, focusing on the role of innate immune cell-derived degrading enzyme MMP8, to shed new light on the processes involved in glycocalyx derangement and subsequent lung damage post-burn.

## 2. Materials and Methods

### 2.1. Patient Enrollment and Sample Collection

For this study’s investigation, serum samples that had been previously assessed for sST2 and IL-33 levels, as well as markers for neutrophil activation, were examined [9,10]. A total of 28 patients and 8 healthy control subjects were enrolled in the study. Patients admitted to the Intensive Care Unit or the Department of Plastic and Reconstructive Surgery at the Medical University of Vienna within 24 h after the burn trauma were included. Further inclusion criteria were an age > 18 years and the presence of an injury covering a minimum of 10% of the total body surface area (TBSA).

### 2.2. Quantification of Serum Analytes via Enzyme-Linked Immunosorbent Assay

The assessment and quantification of serum syndecan 1 (SDC1), syndecan 4 (SDC4), hyaluronan (HA), and matrix metalloproteinase-8 (MMP8) was conducted via enzyme-linked immunosorbent assay (ELISA). The following commercially available ELISA kits were used, adhering to the manufacturers’ instructions: Human Syndecan-1 DuoSet ELISA kit (R&D Systems^®^, Minneapolis, MN, USA, cat#: DY2780); Human Syndecan-4 DuoSet ELISA kit (R&D Systems^®^, Minneapolis, MN, USA, cat#: DY2918); Hyaluronan DuoSet ELISA kit (R&D Systems^®^, Minneapolis, MN, USA, cat#: DY3614-05); Human Heparan Sulfate ELISA Kit (Invitrogen, ThermoFisher Scientific, Waltham, MA, USA, cat#: EEL199); and Human Total MMP8 DuoSet ELISA kit (R&D Systems^®^, Minneapolis, MN, USA, cat#: DY908). The optical density readings were obtained using a Tecan F50 infinite microplate reader (Tecan Group, Männedorf, Switzerland) and processed using Magellan software version 7.2 (Tecan Group, Männedorf, Switzerland). Analyte concentrations were deduced from external standard curves.

### 2.3. Statistical Analysis

For statistical analysis and visualization of the data, IBM SPSS Statistics version 29.0.0 (IBM, Armonk, NY, USA) and GraphPad Prism version 10.0.3 (GraphPad Software, San Diego, CA, USA) were used. The enzyme-linked immunosorbent assay (ELISA) data were preprocessed with the application of the robust regression and outlier removal (ROUT) method (Q = 1%). To minimize potential bias, the Q coefficient was optimized to balance sensitivity and specificity, ensuring the exclusion of only extreme outliers. Statistical analyses were conducted both with and without outlier removal to confirm result consistency. After the detection and removal of outliers, a Shapiro–Wilk test was performed to evaluate the normality of data distribution. Since the ELISA data did not adhere to Gaussian distribution prerequisites, further analysis was conducted using the nonparametric Kruskal–Wallis tests with Dunn’s multiple comparison tests. Where applicable, two-tailed unpaired *t*-tests were used to assess statistical significance between two groups. For correlation analysis between two metric variables, Pearson’s correlation coefficients were used. Additionally, simple logistic regression analyses and odds ratio calculations were conducted to examine associations between nominal and metric variables. The ELISA results are presented as minimum-to-maximum boxplots, with significant time points (adjusted *p*-value < 0.05) distinctly marked in red.

### 2.4. Single-Cell RNA Sequencing (scRNA-Seq) Analysis

Publicly available single-cell data from the NCBI Gene Expression Omnibus (GEO) database (GSE126060) were retrieved. The data were derived from a study involving a murine burn/tenotomy model. Briefly, the animals received a partial-thickness burn injury covering 30% of their total body surface area (TBSA) on their dorsum, together with an Achilles tendon transection on the hind limb. Tissue samples were collected from the hind at day 0 (no burn/tenotomy) and on days 3, 7, and 21 post-injury. Samples were then subjected to single-cell RNA sequencing analysis [30]. Additionally, single-cell RNA sequencing data from healthy human lung tissue (GSE171524) was obtained from the NCBI Gene Expression Omnibus (GEO) repository.

Computational analysis of single-cell RNA sequencing (scRNA-seq) was conducted using R (version 4.3.1, The R Foundation, Vienna, Austria) and RStudio (version 2023.6.2.0 + 561). The Seurat package (version 4.9.9.9059) was used for analysis, adhering to the developer’s Seurat v5 workflow for exploratory data analysis and quality control [31]. In the data preprocessing stage, Seurat objects were merged into a single object and subjected to quality control. Cells with less than 400 or more than 40,000 unique molecular identifiers (UMIs), expressing fewer than 200 or more than 6000 genes, and cells with over 10% mitochondrial reads were excluded from downstream analysis. In addition, mitochondrial genes and genes with less than three UMI counts per feature were removed from the UMI count matrices.

For data integration, the merged dataset was divided into ‘count’ and ‘data’ layers, followed by normalization and the identification of variable features, as outlined in the Seurat v5 integrative analysis guidelines. Prior to Principal Component Analysis (PCA) for dimensionality reduction, features were scaled and centered. Data integration was performed using the “IntegrateLayers” function with an anchor-based RPCA approach, as per Seurat v5 vignette. The resulting single, integrated, batch-corrected expression matrix formed the basis for all further analyses. Clustering was performed according to the standard Seurat workflow, which included UMAP (Uniform Manifold Approximation and Projection) and Louvain clustering through the “RunUMAP”, “FindNeighbors”, and “FindClusters” functions. The first 25 principal components (PCs) were selected based on scree plot analysis, JackStraw resampling, and ElbowPlot evaluation to maximize variance capture while minimizing noise. A clustering resolution of 0.3 was determined through systematic optimization, including clustering tree visualization and differential expression testing across multiple resolutions (0.1–1.0), ensuring biologically meaningful and transcriptionally distinct clusters. The identification of differentially expressed genes (DEGs) was conducted using the MAST statistical framework [32], incorporated in Seurat’s “FindMarkers” and “FindAllMarkers” functions, focusing on genes expressed in a minimum of 25% of cells in one group.

### 2.5. DNA Microarray and Gene Set Enrichment Analysis

Human Affymetrix U133 Plus 2.0 GeneChip™ data (GSE37069) were retrieved from the NCBI’s GEO database, employing GEOquery version 2.66.0 [33] for the download. Differential gene expression (DEG) analysis was performed via the limma package version 3.54.0 [34] in R (version 4.3.1) using RStudio (version 2023.6.2.0 + 561). For the gene set enrichment analysis, only DEGs between the control and burn groups with a log2fold change > 1 and an adjusted *p*-value of <0.05, following the Benjamini–Hochberg correction method, [35] were considered. These DEGs were functionally annotated using the Enrichr [36] package, accessing several databases, including “GO Biological Process 2023”, “KEGG 2021_Human”, and “Reactome 2022”. Results from Enrichr were refined by filtering terms with a Benjamini–Hochberg adjusted *p*-value below 0.05. These were then ranked based on the “combined score”, which incorporates Fisher’s exact test *p*-values and rank deviations. The enriched terms are illustrated in dot plots, depicting combined score and gene ratios of genes.

### 2.6. Bioinformatics Data Visualization

Bioinformatics analysis results were visualized using the following R packages: Seurat v. 4.9.9.9059 [31], ggplot2 v.3.4.2 [37], EnhancedVolcano v.1.18.0 [38], and scCustomize v.1.1.1 [39].

### 2.7. Cell Culture and MMP8 In Vitro Assay

EpiAlveolar^TM^ 3D Small Airway Human MicroTissues were purchased from MatTek (MatTek Corporation, Ashland, MA, USA, cat#: ALV-100-MM). These commercially available human alveolar tissue models are cultivated at the air–liquid interface and incorporate endothelial cells on the basal side of a microporous PET membrane insert, as well as primary human fibroblasts and alveolar epithelial cells at the apical side. The already differentiated models were shipped to our research facility. The EpiAlveolar^TM^ models were maintained using 5 mL of Alveolar assay/maintenance medium (MatTek Corporation, Ashland, MA, USA, cat#: ALV-100-MM) in the basal compartment and 75 µL of the same media on the apical surface, in accordance with the manufacturer’s instructions. The inserts were placed in 12-well plates for subsequent treatments with activated Recombinant Human MMP8 Protein, CF (R&D Systems^®^, Minneapolis, MN, USA, cat#: 908-MP-010).

Primary human small airway epithelial cells (SAECs) were acquired from Clonetics™ (Lonza Group AG, Basel, Switzerland, cat#: CC-2547) and cultured in SABM™ Basal Medium (Lonza Group AG, Basel, Switzerland, cat#: CC-3119) supplemented with SAGM™ SingleQuots™ Kit Supplements and Growth Factors (Lonza Group AG, Basel, Switzerland, cat#: CC-4124). In addition, primary human pulmonary artery endothelial cells (HPAECs) (Lonza Group AG, Basel, Switzerland, cat#: CC-2530) and human lung microvascular endothelial cells (HMVECs) (Lonza Group AG, Basel, Switzerland, cat#: CC-2527) were obtained. HPAECs were cultured in EBM™-2 Basal Medium (Lonza Group AG, Basel, Switzerland, cat#: CC-3156) supplemented with EGM™-2 SingleQuots™ Kit Supplements and Growth Factors (Lonza Group AG, Basel, Switzerland, cat#: CC-4176), while HMVECs were maintained in EBM™-2 Basal Medium (Lonza Group AG, Basel, Switzerland, cat#: CC-3156) with EGM™-2 MV SingleQuots™ Kit Supplements and Growth Factors (Lonza Group AG, Basel, Switzerland, cat#: CC-4147). These pooled primary cells were treated with Recombinant Human MMP8 Protein, CF (R&D Systems^®^, Minneapolis, MN, USA, cat#: 908-MP-010) at confluency.

Recombinant human MMP8 (rhMMP8) was activated at a concentration of 100 µg/mL using 1 mM *p*-Aminophenylmercuric Acetate (APMA) (Sigma-Aldrich^®^, St. Louis, MO, USA, cat#: 164610-700MG) in a buffer containing 50 mM Tris, 10 mM CaCl_2_, and 150 mM NaCl (pH 7.5). The activation reaction was incubated at 37 °C for 1 h. Following activation, the SAECs were treated with the activated rhMMP8 at a 1.0 ng/µL concentration in SABM™ Growth medium. As part of the experimental design, SAECs were also treated with inactive rhMMP8 (prepared without adding APMA), APMA alone in SABM™ Growth medium, or with the growth medium alone as a control. EpiAlveolar^TM^ 3D Small Airway Human MicroTissues were treated with activated rhMMP8 in an alveolar assay medium supplied by the manufacturer or for control with the alveolar assay medium alone.

### 2.8. Immunofluorescence Staining and Microscopy

Paraffin-embedded sections were first deparaffinized by heating them with a heater fan until the paraffin melted, approximately 10 min, ensuring the slides did not dry out at any point. This was followed by two 10 min incubations in xylene and sequential rehydration through a series of ethanol solutions (96%, 80%, and 30%) for a few seconds each, with two repetitions at each concentration. Finally, the sections were rinsed twice in deionized water (dH_2_O) for one minute each. Antigen retrieval was performed using a citrate buffer method. To prepare a 10mM sodium citrate buffer (pH = 6), 25 mL of Target Retrieval Solution 10× (Dako cat#: S2369) was diluted in 225mL dH2O. The slides were placed in a jar containing 80 mL buffer and heated in a pressure cooker for 20 min, followed by cooling to room temperature for another 20 min. Pre-staining, the samples were washed in Phosphate-Buffered Saline (PBS) twice for five minutes each. The primary Anti-Syndecan-1 antibody (Abcam, Cambridge, UK, cat#: ab128936) was diluted 1:500 in 2% PBS/BSA and 100 µL was added to each pap pen-encircled section and incubated overnight at 4 °C. After overnight incubation at 4 °C, the sections were washed in PBS thrice for five minutes each. Subsequently, they were incubated for 30 min at room temperature in the dark with a red fluorescence secondary antibody. The secondary antibody applied was the Goat anti-Rabbit IgG (H + L) Highly Cross-Adsorbed Secondary Antibody, Alexa Fluor™ 546 (Invitrogen, ThermoFisher Scientific, cat#: A-11035), at a dilution of 1:500 in 2% PBS/BSA. The mixture also included 10% goat serum and DAPI (1:1000) (ThermoFisher Scientific, cat#: 62248). Samples underwent three consecutive five-minute washes in PBS and two five-minute washes in dH_2_O. The sections were then mounted with Aqua Polymount medium (Polysciences, Warrington, PA, USA, cat#: 18606) and stored in the fridge. Fluorescence microscopy was conducted using an OLYMPUS BX63 fluorescence microscope, and images were captured and analyzed with the Olympus cellSens Dimension 2.3 (build 18987) software (Olympus, Shinjuku, Tokyo, Japan).

## 3. Results

### 3.1. Transcriptomics Analysis Reveals Glycocalyx Derangement in Response to Burn Injury

**Figure 1:**
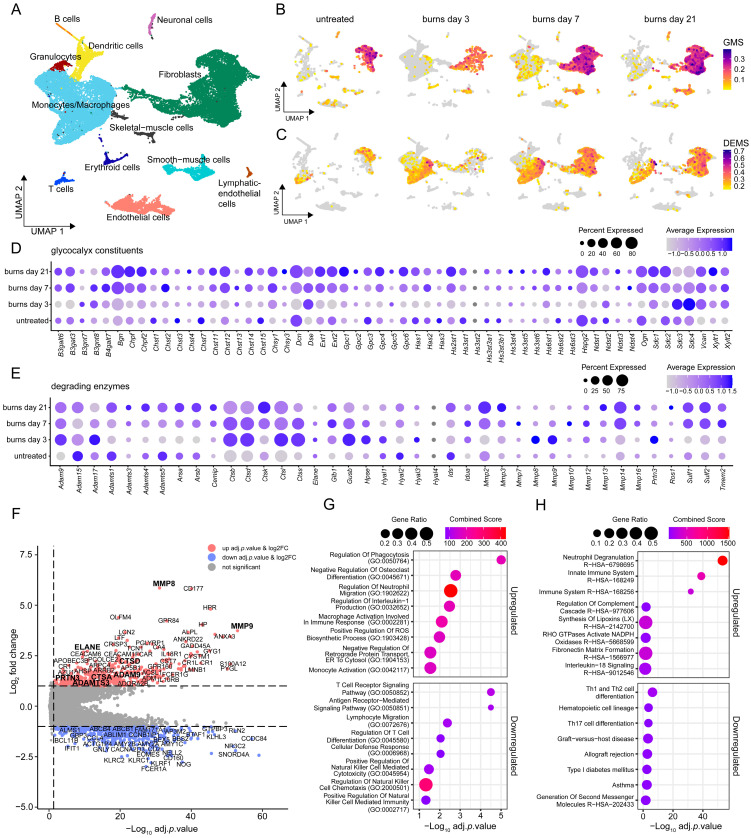
Transcriptomics analysis revealing response of glycocalyx and degrading enzymes post-burn trauma. (**A**) UMAP of comprehensive scRNA-seq analysis identifying 12 unique cell clusters. (**B**) Feature Plots showing a glycocalyx module score (GMS), split by condition. (**C**) Feature Plots illustrating a degrading enzyme module score (DEMS). (**D**) Dot plot of individual glycocalyx constituents used for the GMS module score. (**E**) Dot plot of individual glycocalyx-degrading enzymes used for the DEMS module score. Dot size represents the expression percentage for each gene, with color intensity reflecting average gene expression levels. (**F**) Differentially expressed genes (DEGs) between control and burn groups. Significantly (log2FC > 1, adj. *p* < 0.05) upregulated genes in the burn group show a positive fold change (red), while those downregulated show a negative fold change (blue). (**G**) The top 8 ‘GO Biological Process 2023’ terms associated with significantly upregulated and downregulated DEGs. (**H**) The top 8 terms from a combined query of ‘KEGG 2021 Human’ and ‘Reactome 2022’. Results are displayed as dot plots with their respective combined scores and gene ratios.

To investigate the impact of burn-related trauma on the expression of glycocalyx components and enzymes that are known to or most likely able to degrade the glycocalyx, we re-analyzed a publicly available scRNA-seq dataset of a murine burn injury model [30]. Unbiased clustering analysis identified 12 distinct cell populations, which were further annotated by using established marker genes (Figure 1A and Figure S1A). The module scores for both the expression of glycocalyx- and degrading enzyme-related gene sets were calculated to provide insights into the dynamic changes occurring in response to burn-related trauma. Compared to the untreated condition, a notable decrease in the overall expression of glycocalyx-related mRNAs by day 3 post-injury was observed. This initial decrease was followed by a gradual increase in expression levels, suggesting a dynamic reparative process (Figure 1B). Concurrently, the expression levels of the degrading enzyme module increased on day 3, particularly within the innate immune cell populations (Figure 1C). This coincided with a significant rise in the relative proportion of innate immune cells, as indicated by the monocyte/macrophage cluster expanding from 10.24% to 63.61% and the granulocyte cluster increasing from 0.55% to 4.42% (Figure S1B). Interestingly, these time-dependent responses to trauma were not uniform across all constituents. Some of the glycocalyx components, including *Sdc3* and *Sdc4*, were already increased at this early time point, suggesting an important role in restoring damaged glycocalyx (Figure 1D). By day 7, the expression levels of certain degrading enzymes such as *Adam 17*, *Hpse*, *Hyal1*, *Hyal3*, *Mmp8*, *Mmp9,* and *Prtn3* declined again (Figure 1E). This downregulation contrasted with the progressive increase in certain glycocalyx components, such as *Gpc1*, *Bgn*, and *Vcan*, and most of the glycosaminoglycan biosynthetic enzyme-encoding genes. Notably, this increase was particularly prominent in *Sdc1* compared to the untreated levels. Interestingly, some enzymes like *Adamts3*, *Cemip*, *Ctsk*, *Mmp2*, *Mmp14, Sulf1,* and *Sulf2* exhibited sustained upregulation also at late time points (Figure 1D,E).

To extend the insights from murine scRNA-seq analysis to human systems and enhance translational relevance, we next analyzed transcriptomic data from the “Inflammation and the Host Response to Injury Large-Scale Collaborative Research Program”, including whole blood samples from 244 severely burned patients with >20% TBSA and 35 healthy controls [40]. Differentially expressed genes (DEGs) were calculated between controls and burn patients at various time points post-burn. This analysis revealed the upregulation of 488 genes and the downregulation of 535 genes, fulfilling stringent cutoff criteria of adjusted *p*-value < 0.05 combined with a log2-fold change greater than 1. Several upregulated mRNAs were known to be involved in the regulation of neutrophil migration and activation, such as *CD177*, *MPP1*, *FUT7*, *RAC2*, *FPR2*, *OLFM4*, *ELANE*, *MPO*, *PADI4*, and *ROMO1*. Conversely, genes described to be involved in T cell activation and differentiation, such as *ITK*, *CD3D*, *CD3G*, *ZAP70*, and *MALT1*, were downregulated, demonstrating an altered adaptive immune response following burn injury. Similarly, genes associated with NK cell activity including *CD160*, *KLRC1*, *KLRC2,* and *KLRF1,* among others, were downregulated (Figure 1F). A gene set enrichment analysis of the significantly regulated genes indicated the enhanced activation of innate immune cells post-burn, highlighting processes such as neutrophil activity, phagocytosis, and macrophage activation. Conversely, the enriched terms for downregulated genes were associated with reduced lymphocyte activation, migration, and NK cell-mediated chemotaxis and cytotoxicity (Figure 1G,H). Strikingly, many degrading enzymes, including *MMP8*, *MMP9*, *ADAM9*, *HPSE*, *CTSD*, *CTSG*, *ELANE*, and *PRTN3*, were significantly upregulated also in this data set. Notably, *MMP8* showed the highest overall log2 fold change of 5.86 (Figure 1F). These results corroborate a strong link between innate immune activation and glycocalyx disruption, highlighting MMP8 as a conserved upregulated enzyme that may drive glycocalyx degradation post-burn.

### 3.2. SDC1 and MMP8 Serum Levels Are Increased in Burn Patients

**Figure 2:**
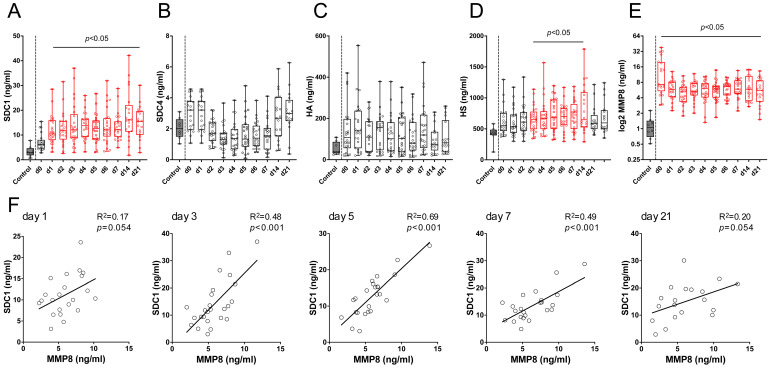
Serum levels of glycocalyx constituents and MMP8 together with SDC1 correlation in burn patients. Systemic levels of SDC1 (**A**), SDC4 (**B**), HA (**C**), HS (**D**), and MMP8 (**E**) were quantified in healthy controls and burn patients over a 21-day follow-up. Data analysis was performed using Kruskal–Wallis tests, complemented by Dunn’s multiple comparisons. Data are presented as minimum to maximum boxplots including all data points. Time points with statistical significance (adjusted *p*-value < 0.05) are marked in red. (**F**) Pearson’s correlation analysis between MMP8 and SDC1 serum levels in burn patients over various time points, with R^2^ values and corresponding *p*-values provided.

To investigate the systemic effect of burn injury on glycocalyx shedding and MMP8 production, we measured the serum levels of SDC1 and 4, hyaluronan (HA), heparan sulfate (HS), and MMP8 in the sera of burn patients over 21 days and healthy controls (Figure 2).

For this study, 28 burn injury patients (7 females, 21 males; mean age 49.6 ± 21.8 years), along with 8 controls (3 females, 5 males; mean age 40.5 ± 19.9 years) were enrolled. An unpaired *t*-test showed no significant age difference between the two groups (*p* = 0.2967). The whole study population demographics are shown in Table 1.

The SDC1 levels were constantly increased from day 1 to day 21 post-burn compared to those in the controls (Figure 2A). The initial mean levels on admission (7.17 ± 3.38 ng/mL) rose to 12.16 ± 5.84 ng/mL on day 1 (adj. *p* < 0.005) and peaked at 17.83 ± 9.98 ng/mL on day 14 (adj. *p* < 0.0001). The SDC1 levels remained elevated at 15.01 ± 6.72 ng/mL on day 21. In contrast, the HA and SDC4 serum levels showed a lower increase, without reaching statistical significance (Figure 2B,C). Specifically, the SDC4 levels increased at early time points (day 0 and 1), which is in line with our bioinformatics data, indicating a rapid increase in the SDC4 expression levels at day 3. Consistent with our bioinformatics findings of an upregulation in glycosaminoglycan biosynthetic enzyme-related genes from day 3 onwards, the heparan sulfate levels in the sera of the burn patients were significantly elevated from day 3 (664.6 ± 202.2 ng/mL) to day 14 (814.3 ± 382.0 ng/mL) (Figure 2D). Interestingly, while there was no consistent correlation of the SDC1 serum levels with the Abbreviated Burn Severity Index (ABSI) or the total body surface area (TBSA) and no significant correlation with third-degree burns over the 21-day period, inhalation injury showed increased odds with elevated SDC1 serum levels in the early phase post-injury. On the day of admission, the odds ratio (OR) for elevated SDC1 was 1.91 (*p* = 0.001). This elevated risk remained significant from day 1 (OR = 1.47, *p* = 0.002) until day 5 (OR = 1.31, *p* = 0.009) (Table 2).

Having identified MMP8 as a significantly upregulated enzyme potentially contributing to glycocalyx disruption through our bioinformatic analysis, we assessed its systemic presence in our study group (Figure 2E). The ELISA measurement of the MMP8 serum levels revealed significantly elevated mean levels in burn patients on the day of admission (13.56 ± 10.94 ng/mL, adj. *p* < 0.001) compared to healthy controls (1.14 ± 0.55 ng/mL), despite the high initial variability. Notably, the MMP8 serum levels remained significantly elevated throughout the 21-day follow-up period (adj. *p* < 0.01). Given the significant increase in the SDC1 levels, simple linear regression analyses were performed to correlate the SDC1 and MMP8 levels post-burn injury (Figure 2F). While the initial correlation on day 1 was weak (R^2^ = 0.17, *p* = 0.054), a significant correlation was observed on day 3 (R^2^ = 0.48, *p* < 0.001). This correlation became even more pronounced on day 5 (R^2^ = 0.69, *p* < 0.001) and remained significant on day 7 (R^2^ = 0.49, *p* < 0.001). These findings suggest that glycocalyx derangement post-burn is a complex process involving both shedding from direct lung injury and systemic degradation by various enzymes. SDC1 appears more susceptible to burn-induced damage, with the observed increase in MMP8 levels and its correlation with SDC1 indicating a coordinated role in promoting SDC1 shedding.

### 3.3. MMP8 Induces SDC1 Shedding in Alveolar Epithelial Cells but Not in Lung-Derived Endothelial Cells In Vitro

**Figure 3:**
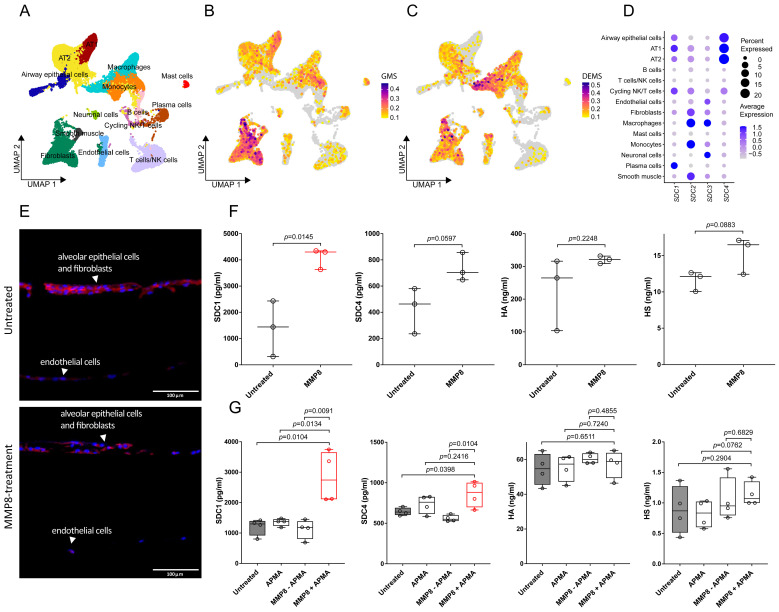
Effects of MMP8 treatment on pulmonary glycocalyx in vitro. (**A**) UMAP representation of scRNA-seq analysis from healthy human lung tissue, identifying 14 distinct cell clusters. (**B**) Feature Plot displaying the glycocalyx module score (GMS) and (**C**) degrading enzyme module score (DEMS), calculated based on the gene sets outlined in Supplementary Figure S2 (Figure S2B,C). (**D**) Dot plot showing syndecan gene expression, with dot size indicating the percentage of expressing cells and color intensity reflecting average expression levels. (**E**) Immunofluorescence imaging of untreated and rhMMP8-treated EpiAlveolar™ 3D lung models, incorporating alveolar epithelial cells (EpiCs), fibroblasts (FBs), and endothelial cells (ECs). The 20× magnification images display DAPI-stained nuclei (blue) and SDC1 fluorescence (red). (**F**) ELISA-based quantitative analysis of SDC1, SDC4, and HA in EpiAlveolar™ model supernatants, with significant differences (*p* < 0.05) marked in red. (**G**) SAECs treated with APMA, rhMMP8 alone, and activated rhMMP8, with SDC1, SDC4, and HA levels in supernatants analyzed via ELISA. Statistical significance was assessed using two-tailed unpaired *t*-tests and the Kruskal–Wallis test followed by Dunn’s multiple comparisons, with significant results (adj. *p* < 0.05) highlighted in red.

To elucidate the cell-type-specific expression of glycocalyx components as a potential target of MMP8 activity in the lung, we analyzed single-cell RNA sequencing data from healthy human lungs [40] (Figure 3A). Cell cluster assignments (Figure S2A), along with comprehensive lists of glycocalyx/GAG-biosynthetic (Figure S2B) and -degrading (Figure S2C) genes, are provided in Supplementary Figure S2. Our analysis revealed the strong expression of key glycocalyx constituents in alveolar epithelial cells, particularly alveolar type II (AT2) and type I (AT1) cells, as well as in fibroblasts (Figure 3B), whereas glycocalyx-degrading enzymes were predominantly found in innate immune cell populations (Figure 3C). Notably, SDC1 and SDC4 were primarily expressed within the alveolar epithelium, with less expression observed in the endothelial and fibroblast compartments. By contrast, the SDC2 and SDC3 transcripts were most abundant in endothelial cells, fibroblasts, and smooth muscle, underscoring the diverse syndecan expression profiles across lung cell types (Figure 3D). To further clarify the cellular source of the shed glycocalyx components, we analyzed the supernatants from alveolar epithelial cells (SAECs) and compared them to those from pulmonary arterial (HPAECs) and microvascular (HMVECs) endothelial cells. Notably, the SAECs released significantly higher levels of SDC1, SDC4, and HA upon MMP8 treatment, whereas the HPAECs and HMVECs showed few if any glycocalyx components in the supernatant (Figure S3), corroborating our bioinformatics data.

To test the hypothesis that MMP8 could directly cleave SDC1 within the lung, EpiAlveolar™ human airway 3D tissue models were treated with recombinant human MMP8. Immunofluorescence staining of SDC1 in lung equivalents revealed a reduced presence of SDC1 and morphological changes in the alveolar barrier (Figure 3E). The quantification of the SDC1, SDC4, HA, and HS levels in the supernatants of the MMP8-treated 3D models via ELISA revealed that the SDC1 levels were significantly higher after the MMP8 treatment (4091 ± 397.3 pg/mL) compared to the untreated condition (1395 ± 1059 pg/mL, *p* = 0.015). Notably, the SDC4 levels also increased in the treated group (735.9 ± 108.1 pg/mL) compared to in the controls (426.0 ± 175.4 pg/mL), though this increase did not reach statistical significance (*p* = 0.0597). Similarly, the HS levels also rose following the MMP8 treatment (15.35 ± 2.5 ng/mL vs. 11.62 ± 1.4 ng/mL in untreated samples), narrowly missing statistical significance (*p* = 0.0883). The HA levels did not significantly differ, aligning with previous findings (Figure 3F). Focusing on the effect of MMP8 on alveolar epithelial cells, neither inactive MMP8 nor its activator *p*-aminophenylmercuric acetate (APMA) alone led to significant SDC1 shedding. However, the mean SDC1 levels more than doubled in the activated-MMP8 (APMA + MMP8)-treated cells (2836 ± 845.6 pg/mL) compared to the untreated condition (1204 ± 274.5 pg/mL, *p* = 0.010). Despite a slight elevation in the HS levels following the MMP8 treatment (1.141 ± 0.2 ng/mL vs. 0.885 ± 0.4 ng/mL in controls), the variability across the samples rendered the change statistically non-significant. Interestingly, while the HA levels in the supernatant of the human small airway epithelial cells (SAECs) remained largely unchanged following recombinant MMP8 treatment, the SDC4 levels were significantly higher in the active-MMP8-treated cells (860.2 ± 158.6 pg/mL) compared to those in the untreated cells (643.9 ± 46.6 pg/mL, *p* = 0.0398) (Figure 3G). These in vitro findings indicate that MMP8 primarily facilitates SDC1 derangement, but also contributes to SDC4 shedding, though to a lesser extent.

## 4. Discussion

Derangement of the glycocalyx plays a significant role in the complications observed in burn patients [22,41], with increasing evidence suggesting that disruptions of the glycocalyx play a role in ARDS development [22,27]. Our analysis showed a complex dynamic between the glycocalyx components and degrading enzymes following burn injury. Initially, mRNAs encoding glycocalyx components and biosynthetic enzymes were transiently downregulated, whereas those encoding degrading enzymes were upregulated, indicating a dynamic interplay between glycocalyx remodeling and immune activation. However, a varied response was observed, with certain glycocalyx components, including *Bgn*, *Gpc1*, *Gpc4*, and, notably, *Sdc1*, showing increased expression levels over time. Interestingly, the glycocalyx module exhibited increased expression primarily in the fibroblast population, while expression in the endothelial cells remained largely unaffected. Given that endothelial glycocalyx damage is common after burns and degrading enzymes were upregulated in the innate immune cells, which first contact the endothelium, this suggests that the endothelium is particularly vulnerable to glycocalyx derangement. Interestingly, our scRNA-seq analysis of healthy human lung tissue reveals that SDC1 and SDC4 are predominantly expressed in alveolar epithelial cells, with lower expression levels detected in endothelial cells, which is consistent with our in vitro observations that, in contrast to pulmonary endothelial cells, MMP8 was only able to shed SDC1 from SAECs. Although our primary focus was on MMP8-mediated epithelial shedding, shedding from other cell types, particularly of cells at the injury site, may also contribute to elevated serum SDC1 [41].

Notably, the in vitro models used in this study capture the acute phase of MMP8-induced glycocalyx shedding, mirroring early post-injury inflammatory responses rather than long-term remodeling. Thus, future studies should explore the lasting impact of sustained enzymatic activity on pulmonary glycocalyx integrity. While there are conflicting reports on neutrophil migration following burn injury, with some studies suggesting impairment [42,43] and others indicating enhanced recruitment [44,45], our analysis showed an upregulation of genes involved in monocyte, macrophage, and neutrophil activity and migration. Notably, the infiltration of neutrophils into the alveoli is a hallmark of ARDS [46], directly impacting mortality [11], where their mediators, including neutrophil elastase [47], among others, lead to increased lung epithelial and endothelial permeability [48]. In this context, we identified *MMP8,* mainly expressed by neutrophils but also macrophages [49], as a significantly upregulated gene post-burn.

The increased expression of *MMP8* after burn-related trauma was systemic and conserved across species, suggesting a critical role for MMP8 in the response to burn-related trauma. Interestingly, MMP8 itself indirectly promotes leukocyte trafficking by cleaving collagen and chemokine-binding proteins [50], including CXCL8 and CXCL5, and enhancing the chemotactic properties of LIX by truncating it at the N-terminus [51]. In our burn patient cohort, we could validate the significantly increased MMP8 serum levels over the duration of the follow-up, suggesting the prolonged activation and degranulation of innate immune cells. Notably, MMP8 correlated with the SDC1 serum levels, and in vitro experiments substantiated that active recombinant MMP8 induces glycocalyx degradation, particularly cleaving syndecan-1. Notably, the activated recombinant MMP8 treatment also resulted in the cleavage of SDC4, suggesting that its catalytic activity is not strictly limited to a specific member of the SDC family.

In line with our observation of MMP8’s role in glycocalyx shedding, it is relevant to note that related MMPs, including MMP2, MMP3, MMP7, MMP9, and MT1-MMP, have been found to cleave SDC1 and SDC4 [25]. Given the interactions between various MMPs and the conserved cleavage sites of syndecans, and also considering their known susceptibility to ADAM [52] and ADAMTS [53] proteinases, our findings reveal that MMP8 also contributes to the shedding of syndecans. Remarkably, MMPs mediate the cleavage of syndecan core proteins, with their associated glycosaminoglycan (GAG) chains remaining intact, which is shown by there being no observed cleavage at the serine residues to which the GAG chains are linked [25]. In murine lung injury models induced by intratracheal lipopolysaccharide (LPS) [54] and bleomycin, [55] researchers have observed the release of long-chain HS into the bronchoalveolar lavage (BAL). The shed GAGs could still be found in the BAL weeks post-injury. The shedding of long chains indicates the cleavage of the proteoglycan anchors, such as SDC1 and SDC4, rather than the HS molecules themselves. Correspondingly, an increase in SDC1 and SDC4 ectodomains has been noted in the mice BAL fluid after LPS-induced injury of the lung, suggesting the cleavage of these proteoglycan anchors. Importantly, in both LPS- and bleomycin-induced injury models, an upregulation of MMPs, particularly MMP9 and, to a smaller degree, MMP2, has been reported [54,55]. However, MMP9 knockout did not mitigate SDC1, SDC4, or HS shedding [54], hinting at compensatory actions by alternate proteases. Burn-induced glycocalyx disruption likely arises from a cocktail of enzymes, including MMP8. To better elucidate MMP8’s specific function, two complementary in vivo strategies are conceivable in future investigations: (i) MMP8-deficient animals, to assess whether its absence can be compensated by other proteases, and (ii) intravenous MMP8 injection, to confirm the enzyme’s capacity to directly induce SDC1 shedding and potentially impact lung or systemic organ integrity. Nevertheless, caution must be exercised when translating findings from murine models to human disease, as genomic responses in mice can differ from those of humans [40]. Future studies should validate our observations in human burn cohorts and, ultimately, in clinical trials evaluating selective MMP8 inhibition. Such translational approaches will be essential for confirming whether MMP8 plays a pivotal role in human glycocalyx derangement and ARDS development post-burn.

We observed that MMP8 preferentially shed SDC1, and, to a lesser extent, SDC4, but not HA, in vitro. This may be due to the specific structural features and cleavage sites present in the syndecan core proteins that make them more susceptible to MMP8’s proteolytic activity. This observation aligns with our finding that the SDC1 serum levels were significantly elevated post-burn, suggesting that systemic glycocalyx derangement preferentially affects SDC1. Despite not reaching statistical significance, the SDC4 levels also markedly increased early post-injury in the burn patients’ sera, consistent with bioinformatics data showing a significant rise in mRNA expression by day 3.

Notably, inhalation injury was associated with elevated SDC1 serum levels in the early phase post-burn. Interestingly, the SDC1 serum levels remained significantly elevated in the burn patients compared to the healthy controls over the 21-day follow-up period, suggesting the involvement of processes beyond direct lung damage, such as systemic inflammation and subsequent shedding caused by immune cell-derived enzymes. The exact mechanisms by which shed SDC1 crosses the alveolar–capillary barrier remain unclear and merit future investigation. Potential routes include increased permeability, transcellular transport, and lymphatic drainage. Notably, in lung injury models such as burns and inhalation trauma, the compromised barrier facilitates SDC1 entry into the bloodstream, as demonstrated by porcine studies linking endothelial damage to elevated SDC1 in both bronchoalveolar lavage and serum [45], and clinical studies associating inhalation injury with endotheliopathy and increased SDC1 shedding [46].

Beyond burn trauma, glycocalyx degradation is a recurring mechanism in diverse pulmonary conditions. In pulmonary fibrosis, syndecan-1 overexpression and shedding were found to contribute to neutrophil chemotaxis, impair alveolar epithelial repair, and drive fibrosis [56] through TGFβ and Wnt signaling [57], highlighting a common pathological mechanism between the two conditions. Similarly, in the context of cardiopulmonary bypass, elevated MMP levels contribute to glycocalyx degradation and endothelial injury [58]. Notably, in coronary artery bypass graft surgery, mechanical ventilation strategies have been shown to reduce MMP8 and MMP9 levels and improve postoperative oxygenation, suggesting a potential reduction in pulmonary glycocalyx damage through the mitigation of MMP-mediated glycocalyx disruption [59]. Additionally, doxycycline, an MMP inhibitor independent of its antimicrobial properties, has demonstrated efficacy in reducing MMP7 and MMP8 levels, thereby decreasing the systemic inflammatory burden and potentially preventing secondary myocardial infarctions [60].

As burn patients with inhalation injury are already at high risk for ARDS [61], MMP8 may further worsen the condition by promoting continuous SDC shedding. MMP-mediated alveolar epithelial glycocalyx derangement has been shown to increase lung permeability, disrupt barrier function [55], and impair surfactant function [29], leading to reduced lung compliance and micro-atelectasis. These disruptions not only compromise the alveolar–capillary integrity but also hinder lung recovery by sequestering growth factors essential for tissue repair, complicating recovery [62,63]. Notably, the extracellular domain of SDC1 plays a critical role in lung repair by influencing cell migration and adhesion through the modification of α2β1 integrin affinity in the lung epithelium [64].

Considering these findings, MMP8-mediated glycocalyx shedding may contribute to ARDS post-burn and impede epithelial recovery after inhalation injury. An optimal ARDS treatment strategy would involve reducing immune cell mobilization and activation while keeping defense intact [46]. Selectively targeting MMP8 could allow for reduced epithelial glycocalyx disruption, while preserving host defense, thereby reducing pulmonary complications in burn patients. Despite advancements in mechanical ventilation strategies, proning, and fluid management, ARDS mortality remains high [65]. With no effective pharmacologic treatments that target the underlying pathology [66], MMP8 inhibition may offer a novel targeted intervention by directly preventing glycocalyx breakdown and subsequent surfactant dysfunction [29]. This approach could theoretically operate in tandem with current standard-of-care interventions, potentially improving outcomes for burn patients facing ARDS. Notably, the administration of the broad-spectrum MMP inhibitor doxycycline has also been shown to mitigate glycocalyx shedding and improve lung function in bleomycin-induced lung injury [55]. In addition, the inhibition of MMPs has been found to attenuate pulmonary damage in endotoxin-induced ARDS in mice [67,68]. While targeting MMPs seems to be a promising therapeutic approach [69], it is important to consider that their role, both in alveolar damage and repair [70], depends on the specific context. Further research is needed to clarify the specific roles of individual MMPs, such as MMP8, in post-burn lung injury. This deeper understanding will aid in understanding pulmonary glycocalyx derangements in burn patients and help in the development of therapeutic strategies, such as targeted MMP inhibition.

## 5. Conclusions

In conclusion, we have demonstrated that burn injury induces the upregulation of various glycocalyx-degrading enzymes, with MMP8 standing out due to its significant and conserved regulation across species. MMP8 emerges as a contributor to the disruption of glycocalyx components post-burn, particularly through SDC1 shedding. These findings suggest that MMP8-mediated glycocalyx derangement plays a central role in burn-related complications, including lung damage. Future research should focus on exploring MMP8 inhibition as a targeted therapeutic strategy to preserve glycocalyx integrity, reduce respiratory and systemic complications, and improve recovery outcomes in burn patients.

## Figures and Tables

**Table 1:** Study population demographics.

Variable	Burn Patients	Controls
n	28	8
Age (years)	49.6 (42.5) ± 21.8 [32.25–72.75]	40.5 (36) ± 19.9 [23–55.75]
F:M ratio (%)	7:21 (25:75)	3:5 (37.5:62.5)
ABSI	7.7 (8) ± 2.8 [5–9]	
APACHE 2	18.4 (18) ± 8.3 [11.5–26.5]	
LOH (days)	41.1 (33) ± 34.0 [15–67.75]	
SAPS II	38.3 (35) ± 16.9 [24.75–48.5]	
SAPS III	31.7 (31.5) ± 10.8 [23–37.25]	
TBSA (%)	32.5 (30) ± 20.2 [16.25–39.50]	
Deceased (%) *	3 (10.7)	
Inhalation injury *	6 (21.4)	
3rd-degree burn (%) *	19 (67.9)	

F:M = female-to-male ratio, ABSI = Abbreviated Burn Severity Index, APACHE 2 = Acute Physiology and Chronic Health Evaluation II (APACHE 2), LOH = Length of Hospitalization, SAPS II/III = Simplified Acute Physiology Score II/III, TBSA = total body surface area, LOH = Length of Hospitalization. Indicated is mean (median) ± SD [interquartile range]. * Indicated is n (SD).

**Table 2:** Correlations between SDC1 and clinical features.

Days After Burn	ABSI			Inhalation Injury			TBSA			3rd-Degree Burn		
	Pearson’s r	95% CI	*p*-Value	OR	95% CI	*p*-Value	Pearson’s r	95% CI	*p*-Value	OR	95% CI	*p*-Value
**0**	0.37	−0.07–0.69	0.097	**1.91**	**1.25–4.16**	**0.001**	0.17	−0.28–0.56	0.462	1.23	0.90–1.95	0.212
**1**	0.27	−0.15–0.61	0.202	**1.47**	**1.12–2.68**	**0.002**	0.04	−0.37–0.43	0.862	1.19	0.98–1.56	0.087
**2**	**0.62**	**0.29–0.82**	**0.001**	**1.58**	**1.15–2.88**	**<0.001**	**0.54**	**0.18–0.78**	**0.006**	1.15	0.98–1.43	0.106
**3**	**0.50**	**0.13–0.75**	**0.011**	**1.39**	**1.13–2.00**	**<0.001**	0.29	−0.12–0.61	0.161	1.12	0.99–1.35	0.091
**4**	**0.46**	**0.07–0.73**	**0.023**	**1.42**	**1.11–2.17**	**0.002**	0.30	−0.12–0.63	0.158	1.20	1.00–1.55	0.056
**5**	0.29	−0.12–0.62	0.164	**1.31**	**1.06–1.84**	**0.009**	0.28	−0.13–0.60	0.182	1.14	0.96–1.41	0.138
**6**	−0.04	−0.42–0.36	0.860	1.08	0.94–1.24	0.266	0.00	−0.39–0.39	0.991	1.03	0.91–1.20	0.636
**7**	−0.05	−0.45–0.37	0.832	1.11	0.93–1.35	0.250	0.05	−0.37–0.45	0.817	1.04	0.88–1.29	0.662
**14**	0.04	−0.40–0.46	0.874	**1.14**	**1.02–1.33**	**0.022**	−0.09	−0.50–0.35	0.695	0.99	0.89–1.12	0.844
**21**	0.14	−0.33–0.56	0.564	1.16	0.98–1.48	0.090	0,06	−0.40–0.50	0.792	0.95	0.79–1.13	0.588

The associations between SDC1 serum levels and clinical scores and features in burn patients from admission (d0) to the end of follow-up (d21) are shown. Pearson’s correlation coefficients were determined for the correlation between two metric variables, as in r (ABSI/SDC1) and r (TBSA/SDC1). To examine relationships between SDC1 and dichotomous variables (inhalation injury and 3rd-degree burn), simple logistic regression and odds ratio calculations were performed. The table presents the Pearson correlation coefficient (r) or odds ratio (OR), the corresponding 95% confidence interval, and the *p*-value. Values of statistical significance are highlighted in bold.

## Data Availability

All scRNA-seq and Affymetrix datasets analyzed in the present study can be publicly accessed via the GEO repository with the identifiers GSE126060, GSE37069, and GSE171524. Detailed instructions for data preprocessing, integration, and analysis are outlined in the Methods Section, allowing other researchers to replicate our findings. Additionally, the R scripts used for bioinformatic analyses will be provided upon request.

## Supplementary Materials

The following supporting information can be downloaded at https://www.mdpi.com/article/10.3390/biology14030269/s1: Figure S1: Cluster marker expression and cell type frequencies in murine burn-related trauma scRNAseq data. Figure S2: Cell-type-specific expression of cluster markers, glycocalyx components, and biosynthetic enzymes as well as degrading enzymes in healthy human lung. Figure S3: In vitro shedding of glycocalyx components in human alveolar epithelial and endothelial cells.

## Abbreviations

The following abbreviations are used in this manuscript:

ABSI	Abbreviated Burn Severity Index
ADAM	A Disintegrin and Metalloproteinase
ADAMTS	A Disintegrin and Metalloproteinase with Thrombospondin Motifs
APACHE II	Acute Physiology and Chronic Health Evaluation II
ARDS	Acute respiratory distress syndrome
BAL	Bronchoalveolar lavage
DAPI	4′,6-Diamidino-2-Phenylindole
DEG	Differentially expressed gene
ELISA	Enzyme-linked immunosorbent assay
GAG	Glycosaminoglycan
HA	Hyaluronic Acid
HMVEC	Human lung microvascular endothelial cells
HPAEC	Human pulmonary artery endothelial cells
HS	Heparan sulfate
LIX	Lipopolysaccharide-Induced CXC Chemokine
LOH	Length of Hospitalization
MMP	Matrix metalloproteinase
MS	Module score
NETosis	Neutrophil extracellular trap formation
PCA	Principal Component Analysis
RPCA	Robust Principal Component Analysis
ROS	Reactive oxygen species
SAECs	Small airway epithelial cells
SAPS	Simplified Acute Physiology Score
SDC	Syndecan
scRNA-seq	Single-cell RNA sequencing
sST2	Soluble Suppression of Tumorigenicity 2
TBSA	Total body surface area
TGFβ1	Transforming Growth Factor Beta 1
UMAP	Uniform Manifold Approximation and Projection
UMIs	Unique molecular identifiers
