# Supplementary material for: Burn-Related Glycocalyx Derangement and the Emerging Role of MMP8 in Syndecan Shedding
Source: Biology (Basel). 2025 Mar 6;14(3):269. doi: 10.3390/biology14030269 (PMC11940132; doi:10.3390/biology14030269)
Supplement: Supplementary file 1 [file biology-14-00269-s001.zip › Supplementary Figure S1.pdf]

Supplementary Figure S1 (Figure S1)

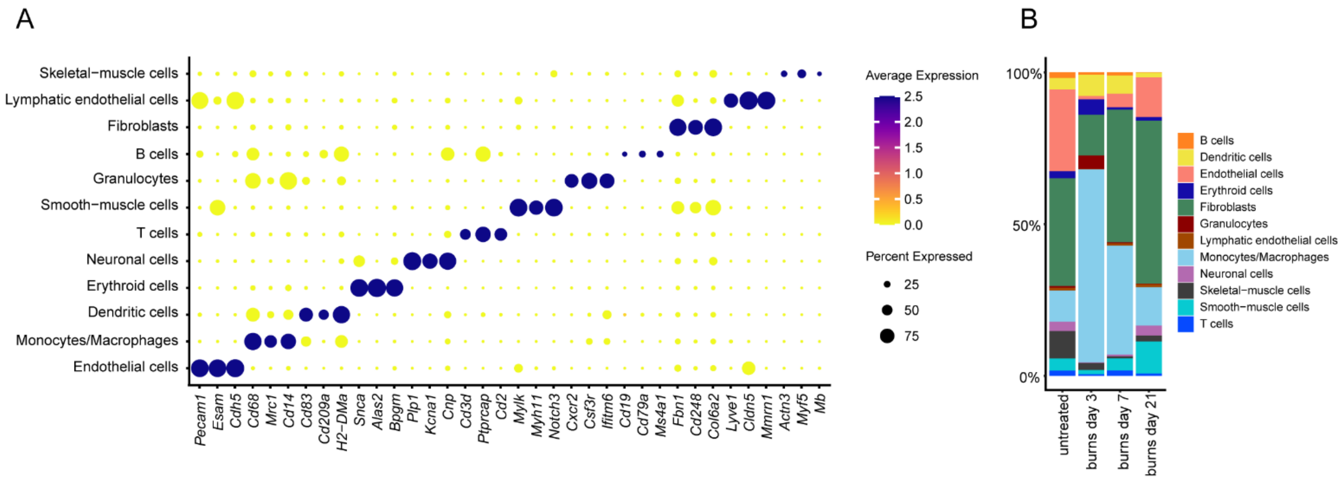

Supplementary Figure S1. Clustermarker expression and cell-type frequencies in murine burn-related trauma scRNAseq data. (A) The left panel shows a dot plot visualization of selected cluster-specific markers, with color shading indicating average gene expression and the size of dots representing the percentage of cells expressing each marker. (B) Stacked bar charts on the right panel illustrate the proportional distribution of each cell cluster across different conditions.
