# Supplementary material for: Burn-Related Glycocalyx Derangement and the Emerging Role of MMP8 in Syndecan Shedding
Source: Biology (Basel). 2025 Mar 6;14(3):269. doi: 10.3390/biology14030269 (PMC11940132; doi:10.3390/biology14030269)
Supplement: Supplementary file 1 [file biology-14-00269-s001.zip › Supplementary Figure S2.pdf]

Supplementary Figure S2 (Figure S2)

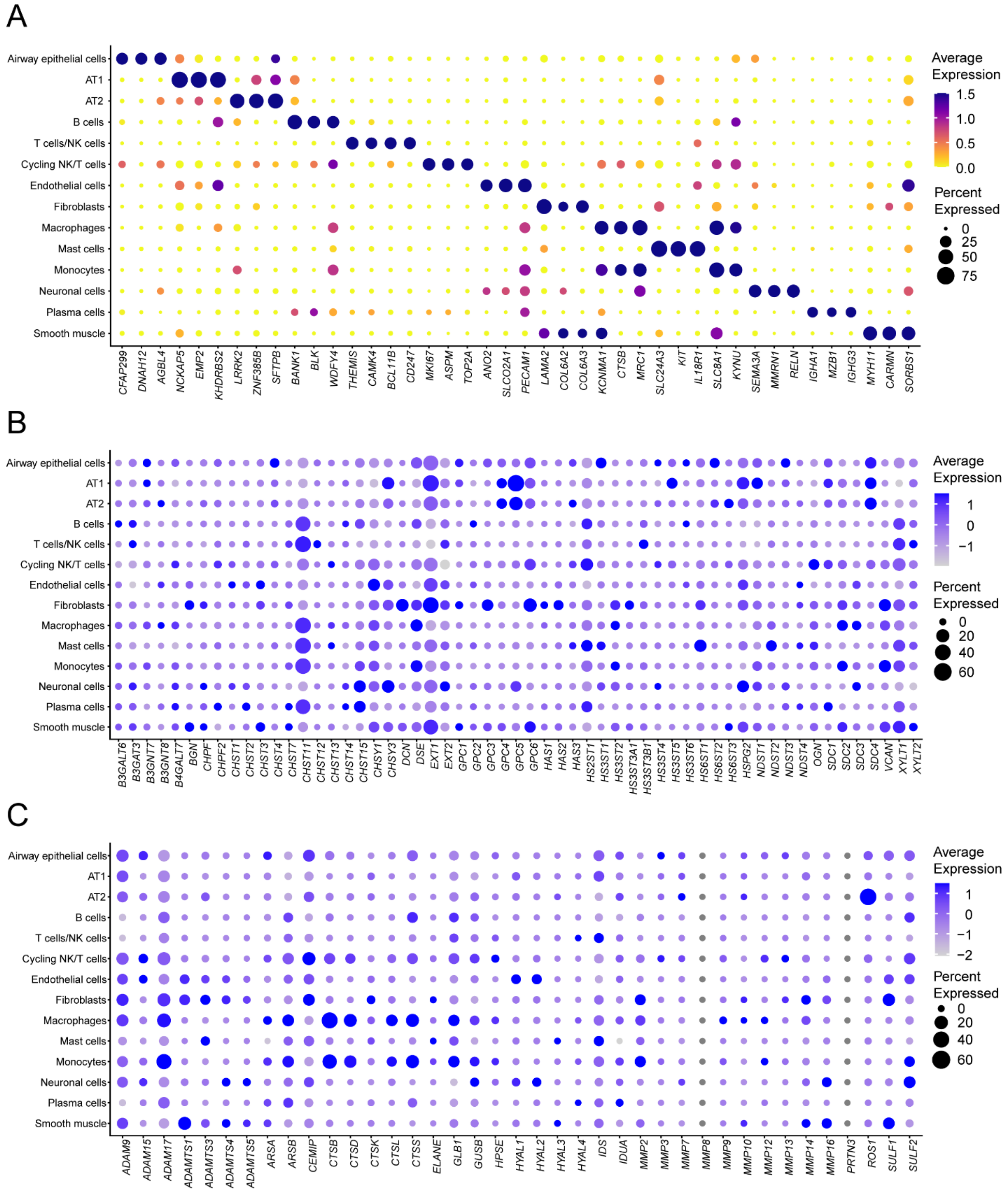

Supplementary Figure S2. Cell-type-specific expression of cluster markers, glycocalyx components and biosynthetic enzymes as well as degrading enzymes in healthy human lung. (A) Dot plot visualization of selected cluster-specific markers, where color shading indicates average gene expression and dot size represents the percentage of cells expressing each marker. (B) Dot plot of individual mRNAs encoding glycocalyx components and glycosaminoglycan biosynthetic enzymes used for the Glycocalyx Module Score (GMS). (C) Dot plot of individual glycocalyx-degrading enzyme-related genes used for the Degrading Enzyme Module Score (DEMS). Dot size represents the expression percentage for each gene, while color intensity reflects average gene expression levels.
