# Supplementary material for: Burn-Related Glycocalyx Derangement and the Emerging Role of MMP8 in Syndecan Shedding
Source: Biology (Basel). 2025 Mar 6;14(3):269. doi: 10.3390/biology14030269 (PMC11940132; doi:10.3390/biology14030269)
Supplement: Supplementary file 1 [file biology-14-00269-s001.zip › Supplementary Figure S3.pdf]

Supplementary Figure S3 (Figure S3)

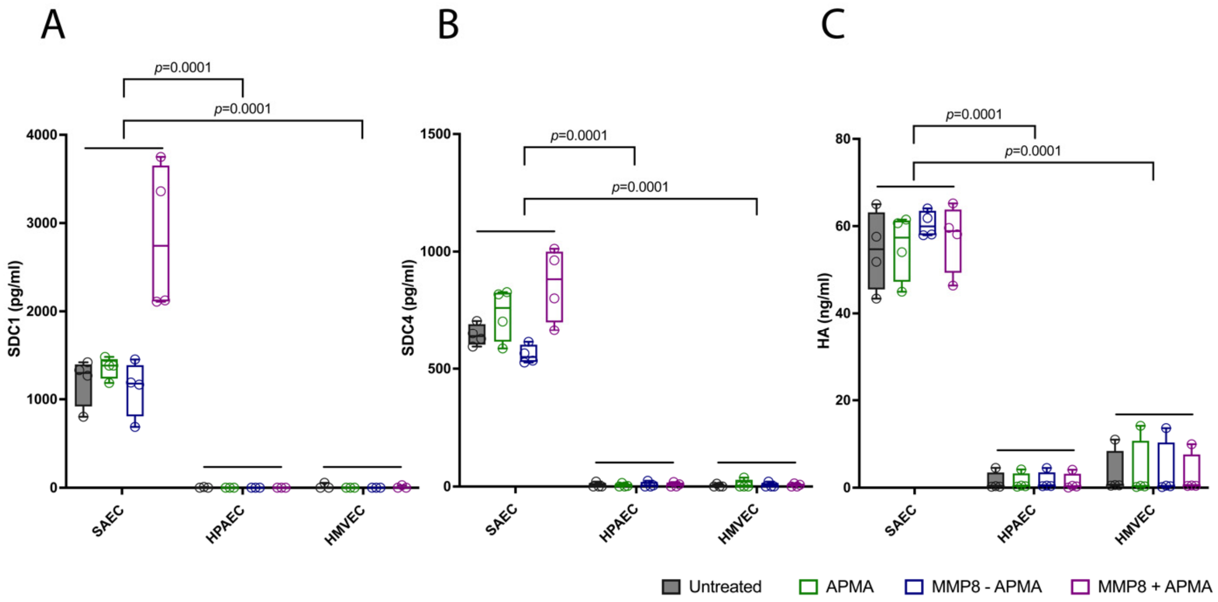

Supplementary Figure S3. In-vitro shedding of glycocalyx components in human alveolar epithelial and endothelial cells. (A) Syndecan-1 (SDC1), (B) Syndecan-4 (SDC4), and (C) Hyaluronic acid (HA) levels in cell culture supernatants of primary human small airway epithelial cells (SAECs), pulmonary artery endothelial cells (HPAECs), and lung microvascular endothelial cells (HMVECs) following different treatments. Data analysis and comparisons between different cell types were performed using Kruskal-Wallis tests, complemented by Dunn's multiple comparisons. Data is presented as minimum to maximum boxplots, including all data points.
